# Effect of silicon carbide on kerf convergence and irregularity of the surface during abrasive water jet machining of fiber-metal hybrid composites

**DOI:** 10.1038/s41598-023-44334-w

**Published:** 2023-10-13

**Authors:** R. Selvam, M. Subramanian, M. Diviya, T. M. Yunus Khan, Rahmath Ulla Baig, Tansir Ahamad, Md. Abul Kalam, Abdul Razak, N. Monish, Anteneh Wogasso Wodajo

**Affiliations:** 1 Department of Mechanical Engineering, St. Joseph’s College of Engineering, Old Mamallapuram Road, Chennai, Tamilnadu 600119 India; 2 Department of Computer Science and Engineering, Amrita School of Computing, Amrita Vishwa Vidyapeetham, Chennai, Tamilnadu 601103 India; 3 Department of Mechanical Engineering, College of Engineering, King Khalid University, 61421 Abha, Saudi Arabia; 4 Department of Industrial Engineering, College of Engineering, King Khalid University, 61421 Abha, Saudi Arabia; 5 Department of Chemistry, College of Science, King Saud University, Riyadh, Saudi Arabia; 6 School of Civil and Environmental Engineering, FEIT, University of Technology Sydney, Ultimo, NSW 2007 Australia; 7 Department of Mechanical Engineering, P. A. College of Engineering (Affiliated to Visvesvaraya Technological University, Belagavi), Mangaluru, 574153 India; 8 Department of Automotive Engineering, College of Engineering and Technology, Dilla University, Dilla, Ethiopia

**Keywords:** Composites, Mechanical properties

## Abstract

The traditional way to machine hybrid composites is hard because they tend to break, have a high retraction, have a high service temperature, and have an uneven surface irregularity. For high-strength fiber/metal composite constructions, alternative machining methods have drawn interest as a solution to these problems. Current research focuses on enhancing the Abrasive Water Jet Machining process by optimizing its variables using a composite material of epoxy reinforced with silicon carbide, stainless steel wire mesh, and Kevlar. The variables assessed are the Nozzle-to-substrate gap (S), the Abrasive discharge molding and different percentages of silicon carbide (SiC) filler (0%, 3%, and 6% by weight), three different types of hybrid laminates (H1, H2, and H3) were produced. The response surface method (RSM) was utilized in this learning, specifically on a central composite design, to calculate and optimize machining variables based on the Kerf convergence ratio (K_t_) and Surface irregularity (R_a_) as responses. According to the results, the traverse feed velocity, Abrasive discharge proportion, and Nozzle-to-substrate gap are the critical factors in determining Surface irregularity and Kerf convergence width (H1 laminate) for a fiber/metal laminate with 0%, 3% and 6% weight fraction. In the case of a 3% weight fraction H2 laminate, the traverse feed velocity was identified as the primary factor affecting the Kerf convergence ratio. In contrast, traverse feed velocity and Nozzle-to-substrate gap had the most significant influence on Surface irregularity. The findings also indicated that S, followed by Abrasive discharge proportion and traverse feed velocity, are the variables that have the most significant influence when cutting 6 wt% SiC filler particle fiber/metal laminate (H3 laminate). For Surface irregularity, the combination of traverse feed velocity and Nozzle-to-substrate gap had the most significant impact. To validate the optimization results, confirmatory tests was conducted, and the findings were very similar to the experimental values, indicating the accuracy and effectiveness of the optimization process. To better understand the manufacturing processes, a scanning electron microscope was used to examine the morphological features of the machined surfaces, such as delamination, fibre breakage, and fibre pull-out.

## Introduction

The demand for fiber-reinforced polymer (FRP) components in modern engineering is high. FRPs can produce almost-final shapes as they transition from soft to hard materials^1–3^. However, advancements in design techniques have made it increasingly necessary to incorporate metal into fiber composites to achieve the optimal working surface or form, resulting in new applications. A novel material called fiber metal laminate (FML) is formed by alternating composite layers and thin metal films^4^. The primary objective of developing fiber metal laminates was to improve impact and fatigue resistance in the aerospace industry^5^. These composites combine metal characteristics, such as exceptional bearing strength and impact resistance, with polymers to overcome the limitations of single monolithic material sheets, such as exceptional fatigue characteristics, high strength, and corrosion resistance^6,7^. Fiber-metal hybrid composites have gained much attention lately because they can combine the stiffness and strength of fibers with the ductility and wear resistance of metals. In recent years, extensive research has been conducted on developing and enhancing these composite materials^8,9^. Several fabrication methods have been explored, including using various types of fibers, metal matrices, and processing methods. Significant research has examined different fiber types and metal matrices and identified factors that enhance mechanical properties. Other crucial areas of research include the development of fabrication techniques, for instance, hot pressing^10^, spark plasma sintering^11^, and hot isostatic pressing^12^. Overall, research on fiber-metal hybrid composites has led to the creation of new and improved composite systems and significant advancements in our understanding of these materials (Table 1).

**Table 1:** Chemical composition of Stainless Steel wire mesh (SS304)^25^.

Grade	C	Mn	Si	P	S	Cr	Mo	Ni	N	Fe
SS304	0.08	1.90	0.70	0.040	0.025	19.0	–	9.0	0.05	69.205

Silicon carbide (SiC), an abrasive and hard substance, is frequently used as a filler in fiber-reinforced composite materials^13^. Adding SiC particles to fiber-metal hybrid composites can affect their stiffness, strength, and fatigue resistance, thereby improving the overall strength of the composite^14^. SiC particles are known to distribute loads more uniformly throughout the composite and reduce stress concentration at the metal-fiber interface, thereby strengthening the metal matrix^8^. SiC particles strengthen the metal matrix and enhance the composite's wear resistance^15^. Due to the abrasive properties of SiC particles, they can shield the metal matrix from deterioration and wear, thus enhancing the composite's resistance to high levels of stress or wear. It is important to note that the mechanical characteristics of fiber metal laminate hybrid composites can be affected by the type of composite system and the quantity of SiC particles used. Therefore, depending on the required qualities of the composite and the manufacturing circumstances, the optimal amount of SiC particles needs to be determined (Table 2).

**Table 2:** Physical properties of SS304 wire mesh^25^.

Grade	Tensile strength (MPa)	Hardness		Yield strength 0.2% proof (MPa)
		Rockwell B (HRB)	Brinell number(HB)	
SS304	520	90	200	210

It is difficult to fully understand the typical machining process of fiber-reinforced composites based on the information available for homogeneous metallic materials due to the non-uniform and directional structure of the microstructure^16^. Nonetheless, significant experimental research has been conducted recently to establish a scientific understanding of the machining of unconventional fiber-reinforced polymers. These studies have shown that Kevlar outperforms other materials because of its exceptional strength-to-weight ratio and fatigue resistance^17^. The alternating hard and soft reinforcement and matrix filler layers in Kevlar fiber-reinforced polymer (KFRP) composites makes conventional machining challenging^18^. Using conventional machining methods to work with Kevlar fiber-reinforced polymer (KFRP) composites can be challenging since the alternating layers of hard and soft materials make it difficult to machine. Conventional machines cannot differentiate between the soft matrix and the rigid reinforcing fiber, which results in tool failure. In order to manufacture aviation components with high precision, it is necessary to use appropriate machining processes. Machining has effectively produced high-quality edge finishes on fiber-metal laminates (FMLs). However, using abrasive glass fibers can cause wear and tear on conventional tools, leading to increased cutting forces, heat generation, and delamination. Diamond-coated mills experience less wear but are expensive and can be quickly damaged by unexpected impacts^19^. Abrasive water jet machining is a highly efficient technique for shaping and cutting high-strength composites, offering precision and accuracy without causing damage to the material. This method is highly valued by manufacturers working with these materials as it allows for intricate shapes and precise cuts (Table 3). Abrasive water jet machining is an exceptional choice for cutting fiber/metal hybrid composites due to its numerous advantages, including its lack of tool wear, cold-cutting process, versatility, reduced environmental impact, and ability to produce components with high strength, stiffness, and durability, making it the preferred choice for manufacturing aerospace or automotive parts.

**Table 3:** Levels of machining variables.

Symbol	Variables	Unit	Level 1	Level 2	Level 3
V_t_	Traverse feed velocity	mm/min	100	300	500
m_a_	Abrasive mass flow rate	g/min	100	200	300
S	Nozzle-to-substrate gap	Mm	1	2	3

Machinability of fiber-metal laminates (FMLs) includes a variety of machining methods, including AWJM, as well as benefits and limits to each approach that critically examines each method^20^. They also discuss the implications of several factors on FML machining, such as abrasive type, abrasive flow rate, standoff distance, and traverse speed^21^. AWJM is also used in the machining of metal matrix composites (MMCs)^22^. Likewise, AWJM is employed for machining fiber-reinforced polymer matrix composites (FRPMCs), a form of fibre-metal hybrid composite^23^. Overall, these literature reviews emphasize the significance of AWJM in machining fibre metal hybrid composites, as well as the need for more studies to optimize the machining process and enhance the quality of the machined components (Table 4).

**Table 4:** Experimental values of K_t_ and R_a_ for Fiber/Metal Hybrid Composites (H1, H2 and H3 laminates).

	Input variables			Output responses					
	Traverse feed velocity V_t_ (mm/min)	Abrasive discharge proportion m_a_ (g/min)	Nozzle-to-substrate gap S (mm)	Kerf convergence ratio (K_t_)			Surface irregularity (R_a_)		
				H1 laminate	H2 laminate	H3 laminate	H1 laminate	H2 laminate	H3 laminate
1	100	100	1	0.84	3.19487	0.9997	11.6	5.36	6.432
2	300	200	1	0.385068	1.77959	1.38686	9.1	5.6	7.254
3	300	100	2	0.575927	2.418	1.54167	8	6.29	7.7367
4	300	200	2	0.45	2.12338	1.78772	8.7	6.36	7.632
5	300	200	2	0.39362	1.99431	1.67494	8.85	6.41	7.8843
6	300	200	2	0.115339	2.15247	1.84578	8.4	6.37	7.4933
7	500	300	1	0.14	0.593197	1.48364	7.5	4.02	5.439
8	500	300	3	0.59	1.492	1.89661	6.73	7.33	9.0159
9	100	300	1	1.48	2.05247	1.64689	11.8	8.63	10.6149
10	300	300	2	0.786877	1.54026	1.8538	8.8	6.33	7.596
11	500	100	3	0.75	1.79088	1.97	5.31	10.25	12.1725
12	500	200	2	0.38389	1.29762	1.84271	6.3	6.72	8.2656
13	100	200	2	0.94805	2.52341	1.75717	10.69	6.28	7.7244
14	300	200	3	0.398461	2.20427	1.95817	7.76	7.01	8.412
15	500	100	1	0.49	1.54468	1.89806	6.25	4.71	5.7933
16	100	100	3	0.197556	2.92	1.67033	9.28	4.52	4.9268
17	300	200	2	0.596018	1.7931	1.83411	8.39	6.19	7.428
18	300	200	2	0.47104	2.16064	1.74168	8.7	6.25	7.6875
19	100	300	3	1.13511	2.21101	2.56742	10.6	5.951	6.48659
20	300	200	2	0.575927	1.87	1.69164	8.87	6.34109	7.359

A mathematical model is created using the response surface approach technique that links the input variables (traverse feed velocity, Abrasive discharge fraction, and nozzle-to-substrate gap) with the output parameters. This model is used to forecast the effect of changing the input variables on the output responses and to find the best values to achieve the intended output responses by exploring the effects of various factors and their complex interactions, which are not possible with other methods which may be found robust providing more detailed analysis of factor interactions. A better surface quality was obtained for adding graphene filler in the hybrid composite with the optimized AWJM process parameters studied using the design of experiments and Taguchi analysis^24^. Response surface graphs were utilized to empirically correlate the impacts of process factors on surface roughness and kerf taper values acquired in experiments, and these values were optimized within the tested range using a desirability technique^25^. Using response surface methodology (RSM), Ramesh et al.^26^ studied how different cooling techniques affected quality attributes while drilling thick composite non-laminates. The application of response surface methodology in abrasive water jet machining has been demonstrated to improve process efficiency and accuracy, which results in decreased machine time, increased productivity, and reduced production costs. Thus optimization of AWJ process parameters is an essential task in machining hybrid composites^27^.

This research investigates the effect of silicon carbide infill weight percentages on the abrasive waterjet processing of hybrid composites incorporating Kevlar fibre and metallic wire mesh. The study investigates how changes in traverse feed velocity, Abrasive discharge proportion, and nozzle-to-substrate gap affect Surface irregularity and Kerf convergence ratio. The research findings indicate that the relationship between these variables is complex, and the optimal choice should be based on specific needs related to Surface irregularity and the Kerf convergence ratio.

## Material and methods

### Hybrid composite preparation with filler

**Figure 1:**
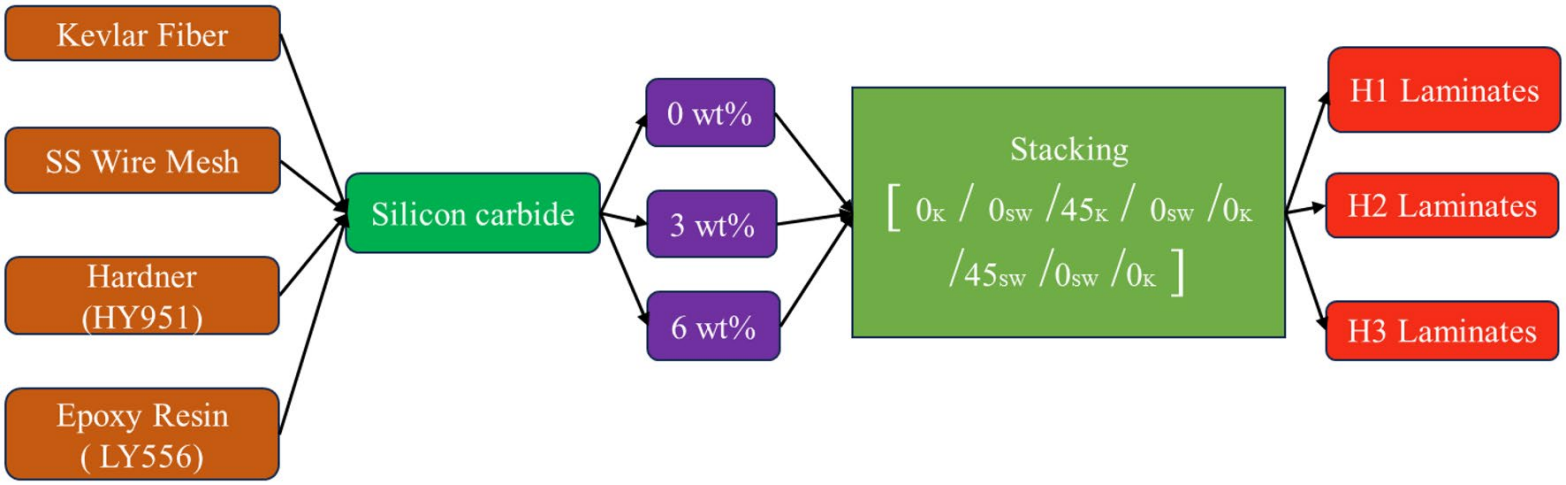
Process flowchart of Abrasive waterjet machining involved in the development of hybrid composites.

Kevlar and SS304 stainless steel wire mesh were reinforcing components to create a hybrid composite material. The matrix of the material was made of a combination of hardener (HY951) and epoxy resin (LY556). The reinforcing materials were 0.2 mm thick and chopped into bits. measuring 300 × 300 mm^2^. To achieve a homogenous mixture, resin and hardener were combined in a 10:1 ratio. Silicon carbide (SiC) was included in the homogeneous matrix mixture as a filler, with weight percentages weighing 0, 3 and 6 wt% SiC. The fabric and stainless steel wire mesh was arranged in a 0°–90° orientation within the die, and the produced matrix mixture was applied to the interface layer using a soft brush. A roller was used to eliminate air pockets, and the composite was laid by hand. A compression molding machine was used to apply 50 bar of pressure to the mold to mould the composite. The same process was used to create hybrid composites with different amounts of SiC filler based on weight. The stacking sequence of the hybrid laminates H1, H2 and H3 are ${[0}^{K}/{0}^{SW}/{45}^{K}/{0}^{SW}/{0}^{K}/{45}^{SW} /{0}^{SW}/{0}^{K}{]}_{s}$. The process flowchart of Abrasive Water Jet Machining (AWJM) involved the development of three different hybrid composites is shown in Fig. 1.

### Abrasive water jet machining of fiber-metal hybrid material

**Figure 2:**
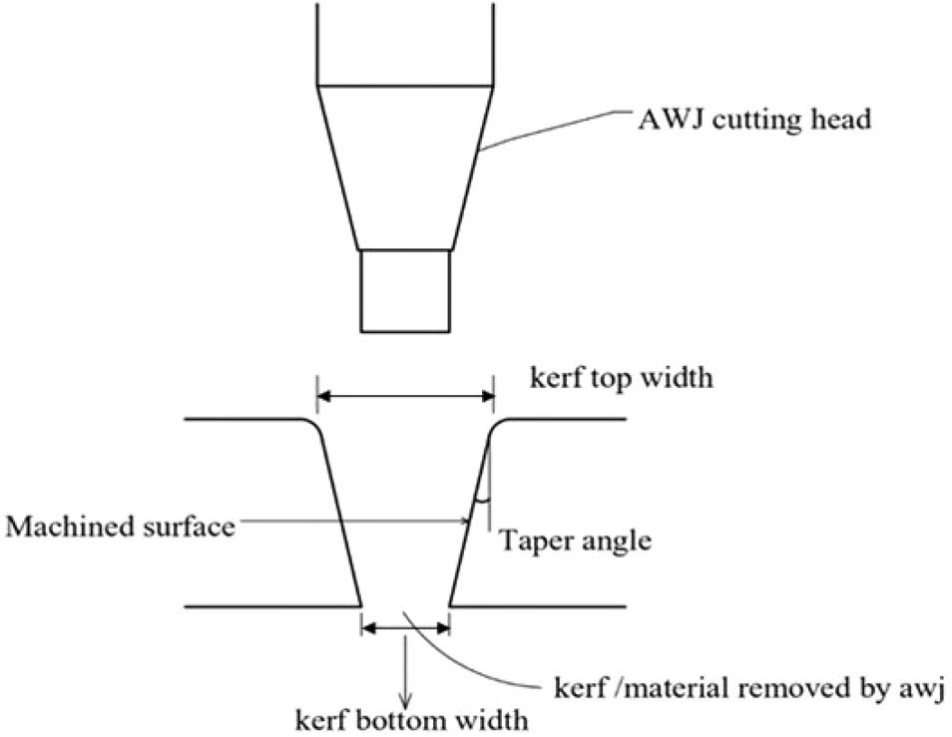
Schematic representation of Kerf convergence ratio.

**Figure 3:**
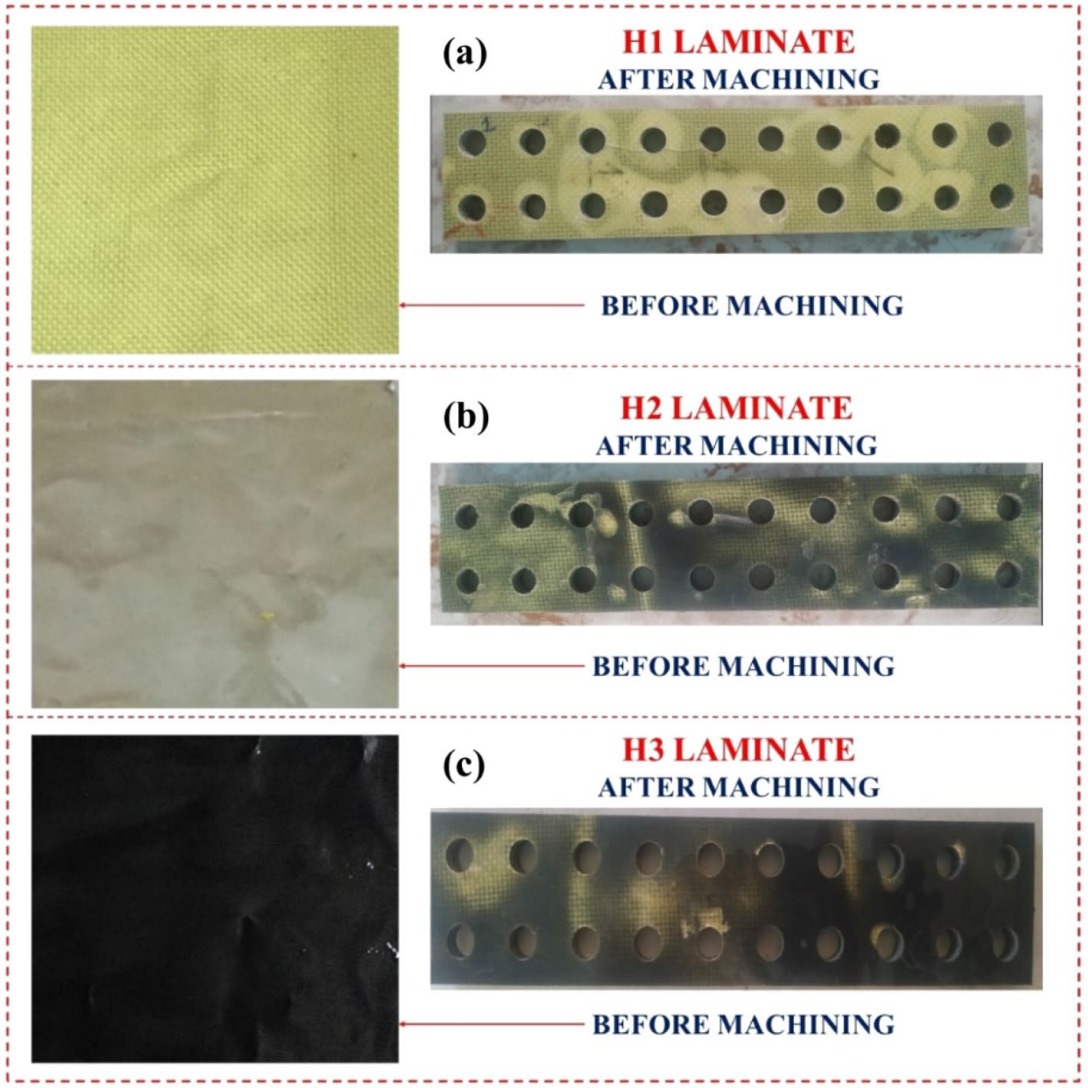
Laminates before and after machining (**a**) H1 laminate (**b**) H2 laminate (**c**) H3 laminate.

The study selected traverse feed velocity, Abrasive discharge proportion, and Nozzle-to-substrate gap as the abrasive water jet machining variables based on a literature review. The OMAX 2626 abrasive water jet machine was chosen for its position precision of 0.002 inches (0.08 mm), straightness accuracy of 0.10 mm/min, and repeatability of 0.050 mm. The machine utilized a 1.25 mm nozzle diameter and a 0.30 mm orifice diameter and employed garnet particles with a mesh size of 80 combined with high-pressure water jets. The study utilized a Central Composite Design for the Design of Experiments (DoE) to conduct experiments to create an L20 orthogonal array with 20 possible experiment combinations. The Surface irregularity of the hybrid composites was determined using MARSURF PS1 with a 5 µm stylus tip radius, and the Kerf convergence width was measured with a video measuring device (OPUS C-2010 made by LEAVE TAIWAN) with a resolution of less than 1 µm as shown in Fig. 2. The laminates before and after machining are shown in Fig. 3.

1 $$Kerf taper \left({K}_{t}\right) (deg)=\frac{\left(Top kerf width -Bottom kerf width\right)x180}{(2\pi \times Specimen thickness)}$$

## Results and discussion

### Mathematical modelling using response surface methodology (RSM)

Response Surface Methodology is an analytical method used to assess the effect of multiple predictor variables on a response variable^24,28–30^. This approach involves creating a mathematical model that predicts the response variable based on different combinations of predictor variables to identify the combination of predictor variable values that optimizes or achieves the desired response^31^. The study utilized Response Surface Methodology to investigate the impact of independent factors such as traverse feed velocity, Abrasive discharge proportion, and Nozzle-to-substrate gap on the response variable. The study results indicated that Response Surface Methodology is an effective approach to optimize the abrasive waterjet machining process. The study used Central Composite Design with three factors and three levels to conduct experiments and develop a mathematical model that depicts the relationship between machining settings and response variables^32,33^.

### Impact of machining variables on Kerf convergence ratio (K_t_) of H1 Laminate

Equation (2) calculates the relationship between the Abrasive discharge proportion and the machining variables of traverse feed velocity, Abrasive discharge proportion, and Nozzle-to-substrate gap for H1 laminate containing 0 wt% SiC filler.

2 $${K}_{t}= +0.833199-0.000636 {V}_{t}+0.003974 {m}_{a}-0.466533 S-0.000013 \times {V}_{t}\times {m}_{a}+0.001061\times {V}_{t}\times S+0.000609\times {m}_{a}\times S$$

Equation (2) indicates that traverse feed velocity and Nozzle-to-substrate gap variables have a negative impact on Abrasive discharge proportion. In contrast, the combination of machining variables positively affects the Abrasive discharge proportion for fiber/metal laminates containing 0% weight SiC filler particles. The 2FI model is preferred for evaluating the interaction between Abrasive discharge proportion, traverse feed velocity, and Nozzle-to-substrate gap. The model is significant, and Table 5 displays the impact and interactions of the machining variables on Abrasive discharge proportion. The R^2^ and modified R^2^ values of 0.9755 and 0.9535 for H1 laminates demonstrate a significant relationship between the variables. The model's *p*-value becomes less than 0.05, further validating its significance for H1 laminates. Traverse feed velocity has the highest impact on the Abrasive discharge proportion at 26.29%, followed by the interaction between traverse feed velocity and Abrasive discharge proportion at 28.36%. The abrasive discharge proportion's effect is 8.51%, 1/3 of the traverse feed velocity. The interaction between traverse feed velocity and Nozzle-to-substrate gap has a moderate effect at 18.75%, almost equal to the individual impact of traverse feed velocity on Abrasive discharge proportion. These findings indicate that traverse feed velocity is critical in determining Abrasive discharge proportion for H1 laminates.

**Table 5:** ANOVA of Kerf convergence ratio (Kt) for Fiber/Metal Hybrid Composites (H1, H2 and H3 laminates).

Kerf convergence ratio (K_t_)	Hybrid Laminate (H1)					Hybrid Laminate (H2)					Hybrid Laminate (H3)				
Source	SS	dof	MS	F-value	*p*-value	SS	dof	MS	F-value	*p*-value	SS	dof	MS	F-value	*p*-value
Model	1.88	9	0.2084	44.30	< 0.0001	6.01	6	1.00	80.55	< 0.0001	1.62	6	0.2702	64.59	< 0.0001
A-Traverse feed velocity	0.5048	1	0.5048	107.31	< 0.0001	3.82	1	3.82	307.48	< 0.0001	0.0202	1	0.0202	4.83	0.0467
B- Abrasive discharge proportion	0.1635	1	0.1635	34.74	0.0002	1.58	1	1.58	127.35	< 0.0001	0.1873	1	0.1873	44.77	< 0.0001
C- Nozzle-to-substrate gap	0.0070	1	0.0070	1.48	0.0251	0.2112	1	0.2112	16.99	0.0012	0.7009	1	0.7009	167.53	< 0.0001
AB	0.5447	1	0.5447	115.79	< 0.0001	0.0452	1	0.0452	3.63	0.0791	0.5162	1	0.5162	123.38	< 0.0001
AC	0.3601	1	0.3601	76.55	< 0.0001	0.1989	1	0.1989	15.99	0.0015	0.1530	1	0.1530	36.56	0.0066
BC	0.0297	1	0.0297	6.32	0.0307	0.1474	1	0.1474	11.86	0.0044	0.0436	1	0.0436	10.43	
Residual	0.0470	13	0.0047			0.1617	13	0.0124			0.0544	13	0.0042		
Lack of Fit	0.0152	8	0.0030	0.4768	0.7822	0.0391	8	0.0049	0.1995	0.9776	0.0286	8	0.0036	0.6914	0.6944
Pure Error	0.0319	5	0.0064			0.1225	5	0.0245			0.0258	5	0.0052		
Cor Total	1.92	19				6.17	19				1.68	19			
R^2^	0.9755					0.9738					0.9675				
*Adj* R^2^	0.9535					0.9617					0.9526				
*Pred* R^2^	0.9085					0.9511					0.9379				

**Figure 4:**
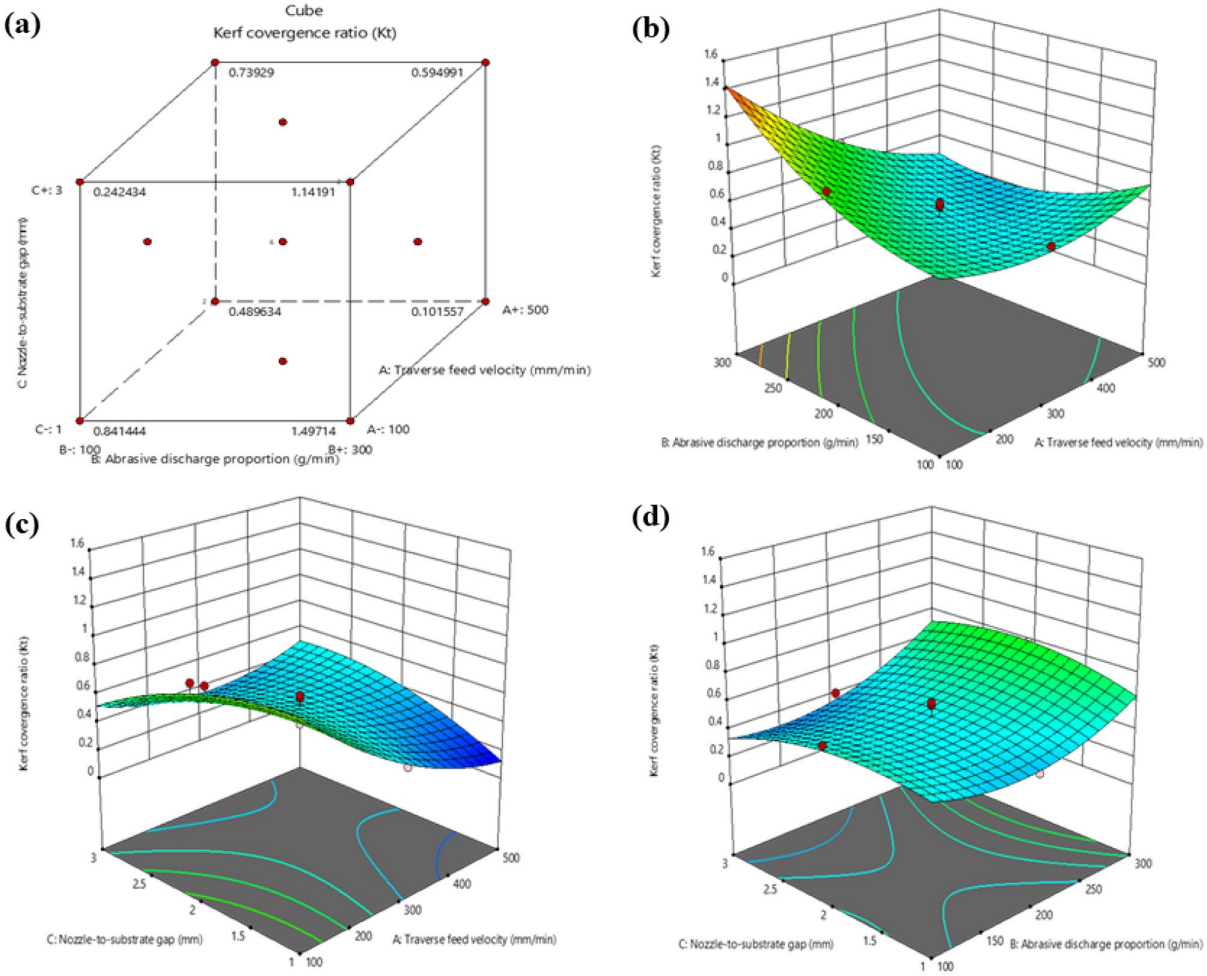
Interaction between Kerf convergence ratio and machining variables (**a**) Cube plot (**b**) 3D surface plot with predictors Abrasive discharge proportion and traverse feed velocity for Kerf convergence ratio (**c**) 3D surface plot with predictors traverse feed velocity and Nozzle-to-substrate gap for Kerf convergence ratio (**d**) 3D surface plot with predictors Abrasive discharge proportion and Nozzle-to-substrate gap for Kerf convergence ratio.

Figure 4 illustrates the impact of various machining variables on the Kerf convergence ratio. As the traverse feed velocity increases for H1 laminates, there is a decrease in the Kerf convergence ratio^34^. This is due to the reduced time the waterjet has to erode the material at the bottom of the cut as the nozzle travels faster over the material being cut, resulting in a smaller kerf at the bottom. Additionally, the abrasive particles in the water jet have increased velocity at the top of the cut, causing more erosion and a wider kerf at the top. Combining these two effects leads to a decrease in the Kerf convergence ratio.

Similarly, the Kerf convergence ratio increases when the Abrasive discharge proportion is increased during waterjet machining of H1 laminates. A higher mass flow rate of abrasive particles produces a more intense cutting action, resulting in increased erosion of the laminate at the extremity of the kerf as more abrasive particles are directed towards the material. This leads to a broader kerf at the bottom of the cut and a higher Kerf convergence ratio. Furthermore, an increase in the mass flow rate of abrasive particles leads to a higher total cutting velocity, causing an increase in the Kerf convergence ratio. However, changing the Nozzle-to-substrate gap in abrasive waterjet machining of H1 laminates does not impact the Kerf convergence ratio. The width of the kerf is unaffected by the distance between the nozzle and the material being cut. The ratio of kerf width at the top and bottom is determined by the erosion rate of the material at those points, which is primarily influenced by the waterjet pressure, traverse feed velocity, and Abrasive discharge proportion. By altering the gap between the nozzle and the material, the Nozzle-to-substrate gap has an indirect impact on the waterjet pressure and cutting velocity. However, it does not have a direct influence on the Kerf convergence ratio.

### Impact of machining variables on Surface irregularity (R_a_) of H1 Laminate

Equation (3) establishes the link between the machining variables of traverse feed velocity and surface irregularity, Abrasive discharge proportion, and Nozzle-to-substrate gap for H1 laminates containing 0 wt% SiC filler.

3 $${R}_{a}= +13.93450-0.014640 {V}_{t}-0.000391 {m}_{a}-1.31887 S+0.00000718750 \times {V}_{t}\times {m}_{a}+0.001131\times {V}_{t}\times S+0.001612\times {m}_{a}\times S$$

Equation (3) indicates that traverse feed velocity, Abrasive discharge proportion, and Nozzle-to-substrate gap variables have a negative impact, while the combination of machining variables has a positive impact on Surface irregularity (Ra) for fiber/metal laminates that contain 0% weight SiC filler particles. The method of choice for examining the correlation among surface irregularity, traverse feed velocity, Abrasive discharge proportion, and Nozzle-to-substrate gap is the 2FI model. The model is significant, and Table 6 presents the significant effect and interactions of the machining variables on Surface irregularity. The R^2^ and adjusted R^2^ values of 0.9904 and 0.9859 for H1 laminates indicate a strong relationship between the output and input response parameters^35^. The *p*-value is less than 0.05 further confirms the significance of the model for H1 laminates. In the case of H1 laminates, traverse feed velocity has the most significant impact on Surface irregularity, accounting for over 80% of the total effect. In contrast, Abrasive discharge proportion and Nozzle-to-substrate gap have minimal impact. The influence of the interactions between traverse feed velocity and Abrasive discharge proportion, Abrasive discharge proportion and Nozzle-to-substrate gap, and traverse feed velocity and Nozzle-to-substrate gap on Surface irregularity is negligible compared to the individual contribution of Abrasive discharge proportion. This indicates that traverse feed velocity is crucial in determining Surface irregularity for H1 laminates^36^.

**Table 6:** ANOVA of Surface irregularity (R_a_) for Fiber/Metal Hybrid Composites (H1, H2 and H3 laminates).

Surface irregularity (R_a_)	Hybrid Laminate (H1)					Hybrid Laminate (H2)					Hybrid Laminate (H3)				
Source	SS	dof	MS	F-value	*p*-value	SS	dof	MS	F-value	*p*-value	SS	dof	MS	F-value	*p*-value
Model	55.46	6	9.24	222.75	< 0.0001	35.02	6	5.84	561.30	< 0.0001	50.23	6	8.37	134.45	< 0.0001
A—Traverse feed velocity	47.87	1	47.87	1153.62	< 0.0001	19.12	1	19.12	50.38	< 0.0001	30.38	1	30.38	32.55	< 0.0001
B—Abrasive discharge proportion	2.49	1	2.49	60.00	< 0.0001	8.63	1	8.63	12.30	0.0039	10.70	1	10.70	7.02	0.0200
C—Nozzle-to-substrate gap	4.32	1	4.32	104.02	< 0.0001	4.54	1	4.54	436.95	< 0.0001	3.68	1	3.68	48.24	< 0.000,
AB	0.1653	1	0.1653	3.98	0.0673	2.07	1	2.07	830.23	< 0.0001	3.00	1	3.00	171.91	< 0.0001
AC	0.4095	1	0.4095	9.87	0.0078	0.5240	1	0.5240	1838.91	< 0.0001	2.03	1	2.03	487.92	< 0.0001
BC	0.2080	1	0.2080	5.01	0.0433	0.1279	1	0.1279	199.01	< 0.0001	0.4373	1	0.4373	59.09	< 0.0001
Residual	0.5395	13	0.0415			0.1352	13	0.0104			0.8094	13	0.0623		
Lack of Fit	0.3160	8	0.0385	0.8837	0.5837	0.1008	8	0.0126	1.83	0.2624	0.6231	8	0.0779	2.09	0.2164
Pure Error	0.2235	5	0.0447			0.0344	5	0.0069			0.1863	5	0.0373		
Cor Total	56.00	19				35.16	19				51.04	19			
R^2^	0.9904					0.9962					0.9841				
*Adj* R^2^	0.9859					0.9944					0.9768				
*Pred* R^2^	0.9584					0.9869					0.9593				

**Figure 5:**
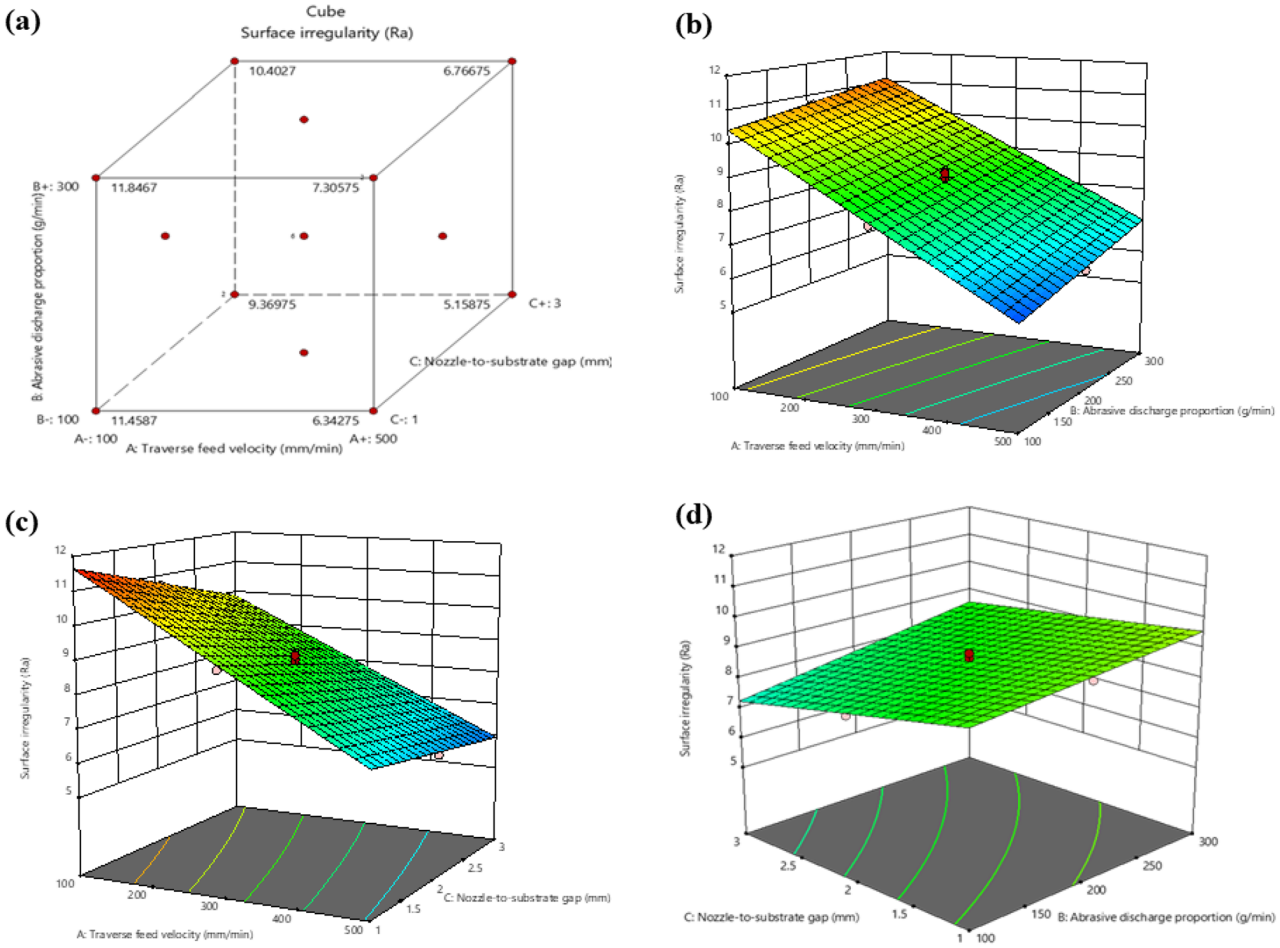
Interaction between Surface irregularity and machining variables **(a)** Cube plot **(b)** 3D surface plot with predictors Abrasive discharge proportion and traverse feed velocity for Surface irregularity **(c)** 3D surface plot with predictors traverse feed velocity and Nozzle-to-substrate gap for Surface irregularity **(d)** 3D surface plot with predictors Abrasive discharge proportion and Nozzle-to-substrate gap for Surface irregularity.

Figure  5 depicts the correlation between Surface irregularity and different machining variables. Figure 5a is a cube plot, a 3D graphical representation of the variation in Surface irregularity across different levels of two input factors in the experimentation. It illustrates a significant influence of the factors on the response variable. Figures 4b–d demonstrate that when the velocity of the traverse feed is raised from 100 to 500 mm/min, Surface irregularity decreases. This is because a higher traverse feed velocity leads to more robust collisions between particles in the jet and more time for cutting. However, suppose the traverse feed velocity is too high. In that case, the abrasive flow rate per unit area increases, resulting in larger delaminations on the machined surface of fiber/metal laminates and a rougher surface irregularity.

Conversely, decreased traverse feed velocity yields a finer finish of the machined surface. Furthermore, it can be observed that when the Nozzle-to-substrate gap is low, and the ma is high, Surface irregularity decreases. This is because as the Nozzle-to-substrate gap increases, the collision force of particles on the workpiece decreases, resulting in the formation of rough peaks on the machined surface.

### Impact of machining variables on Kerf convergence ratio (K_t_) of H2 laminate

Equation (4) calculates the relationship between the Kerf convergence ratio and the machining variables of traverse feed velocity, Abrasive discharge proportion, and Nozzle-to-substrate gap for H2 laminates containing 3 weight percent SiC filler.

4 $${K}_{t}= +4.65694-0.005420 {V}_{t}-0.007821 {m}_{a}-0.362668 S+0.00000375643 \times {V}_{t}\times {m}_{a}+0.000788\times {V}_{t}\times S+0.001358\times {m}_{a}\times S$$

Equation (4) reveals that variable interactions have a positive impact on the Kerf convergence ratio for fiber/metal laminates containing 3 wt% SiC filler particles. In contrast, traverse feed velocity, Abrasive discharge proportion, and Nozzle-to-substrate gap have a negative impact. The preferred method for analyzing the connection between Kerf convergence ratio, traverse feed velocity, Abrasive discharge proportion, and Nozzle-to-substrate gap is the 2FI model. The model is significant, and Table 5 presents the significant effect and interactions of the machining variables on the Kerf convergence ratio. The R2 and adjusted R2 values of 0.9738 and 0.9617 for H2 laminates indicate a strong correlation between the input and output response variables. The model's significance for H2 laminates is further confirmed by a *p*-value less than 0.05. Traverse feed velocity contributes over 50%, and the Abrasive discharge proportion contributes over 25% to the Kerf convergence ratio for H2 laminates, accounting for two-thirds of the total contribution. Other interactions between machining variables have minimal impact on determining the Kerf convergence ratio. Therefore, traverse feed velocity and Abrasive discharge proportion are critical factors in determining the Kerf convergence ratio for H2 laminates.

**Figure 6:**
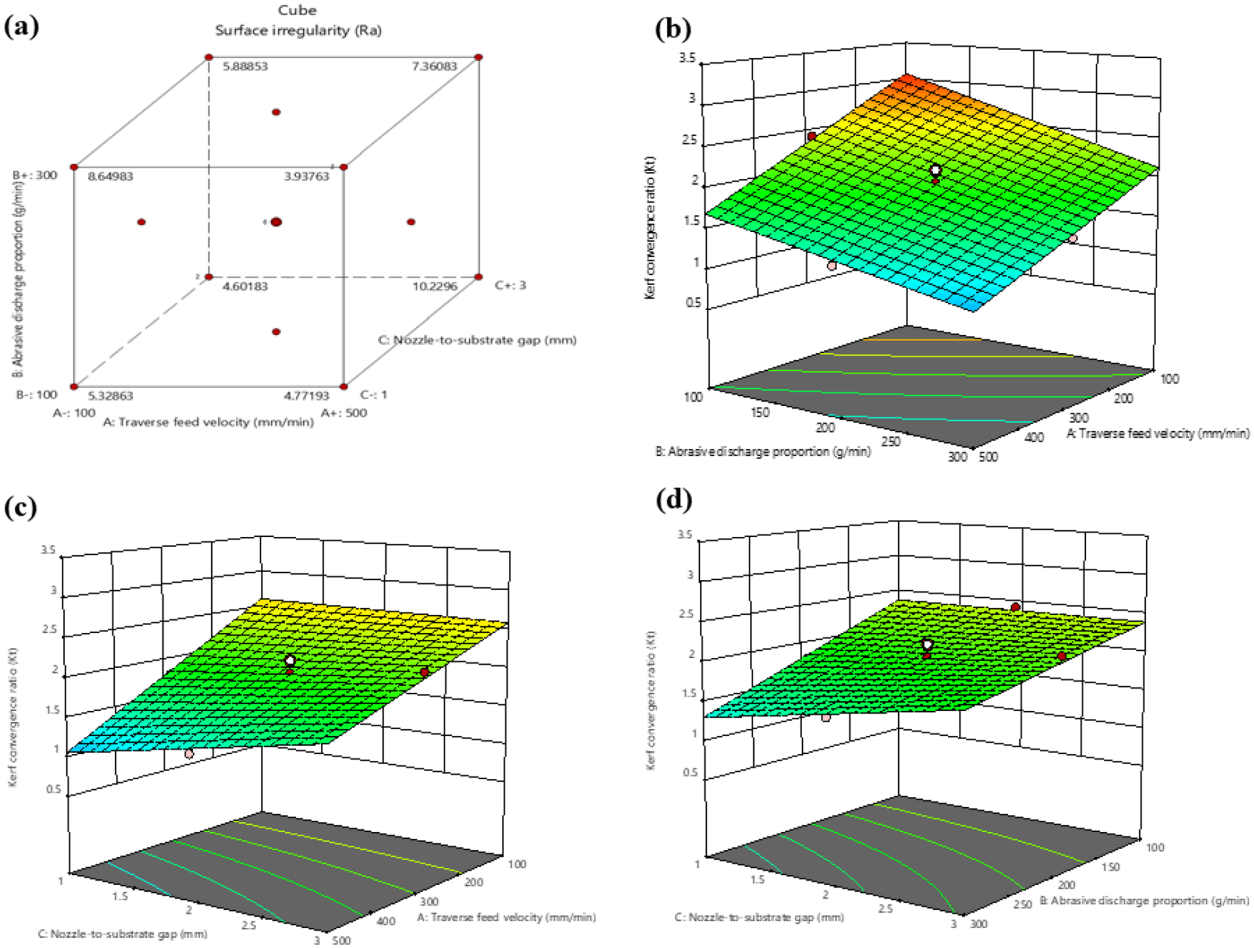
Interaction between Kerf convergence ratio (K_t_) and machining variables **(a)** Cube plot **(b)** 3D surface plot with predictors Abrasive discharge proportion and traverse feed velocity for Kerf convergence ratio **(c)** 3D surface plot with predictors traverse feed velocity and Nozzle-to-substrate gap for Kerf convergence ratio **(d)** 3D surface plot with predictors Abrasive discharge proportion and Nozzle-to-substrate gap for Kerf convergence.

According to Fig. 6, the Kerf convergence ratio reduces as the traverse feed velocity and Abrasive discharge proportion increase. When the traverse feed velocity is higher, the Kerf convergence ratio decreases since the water jet has less time to erode the material, leading to a narrower kerf. Conversely, a lower traverse feed velocity causes more abrasive particles to hit the workpiece, resulting in a wider Kerf convergence ratio. Furthermore, a substantial increase in the Abrasive discharge proportion causes a decrease in the Kerf convergence ratio due to the formation of particle separation at larger widths.

### Impact of machining variables on Surface irregularity (R_a_) of H2 Laminate

Equation (5) determines the relationship between Surface irregularity and the machining variables of traverse feed velocity, Abrasive discharge proportion, and Nozzle-to-substrate gap for H2 laminates containing 3 weight percent silicon carbide filler.

5 $${R}_{a}= +3.91560-0.003928 {V}_{t}+0.026887 {m}_{a}-0.627838 S-0.000052 \times {V}_{t}\times {m}_{a} +0.007731\times {V}_{t}\times S-0.005086\times {m}_{a}\times S$$

In Eq. (5), Abrasive discharge proportion is found to have a positive effect on Surface irregularity. It is the most influential parameter in predicting it, while traverse feed velocity and Nozzle-to-substrate gap have a negative effect for 3 wt% silicon carbide filler particles on fiber/metal laminate. The 2FI model is the preferred model for analyzing the relationship between Surface irregularity, traverse feed velocity, Abrasive discharge proportion, and Nozzle-to-substrate gap, and it is significant. Table 6 shows a substantial effect and strong correlation of machining variables on surface irregularity. For H2 laminate, the model is very strong, with R2 and adjusted R2 values of 0.9962 and 0.9944, respectively. The *p*-value is less than 0.05, reflecting the model's statistical significance for H2 laminates. Traverse feed velocity accounts for more than 60% of the contribution to Surface irregularity, the Abrasive discharge proportion accounts for almost 35%, and the Nozzle-to-substrate gap contributes only a small percentage. The interactions between traverse feed velocity and Abrasive discharge proportion, Abrasive discharge proportion and Nozzle-to-substrate gap, and traverse feed velocity and Nozzle-to-substrate gap have little impact on Surface irregularity compared to the individual contributions of traverse feed velocity and Abrasive discharge proportion. This indicates that traverse feed velocity and Abrasive discharge proportion are the key factors in determining Surface irregularity for H2 laminate.

**Figure 7:**
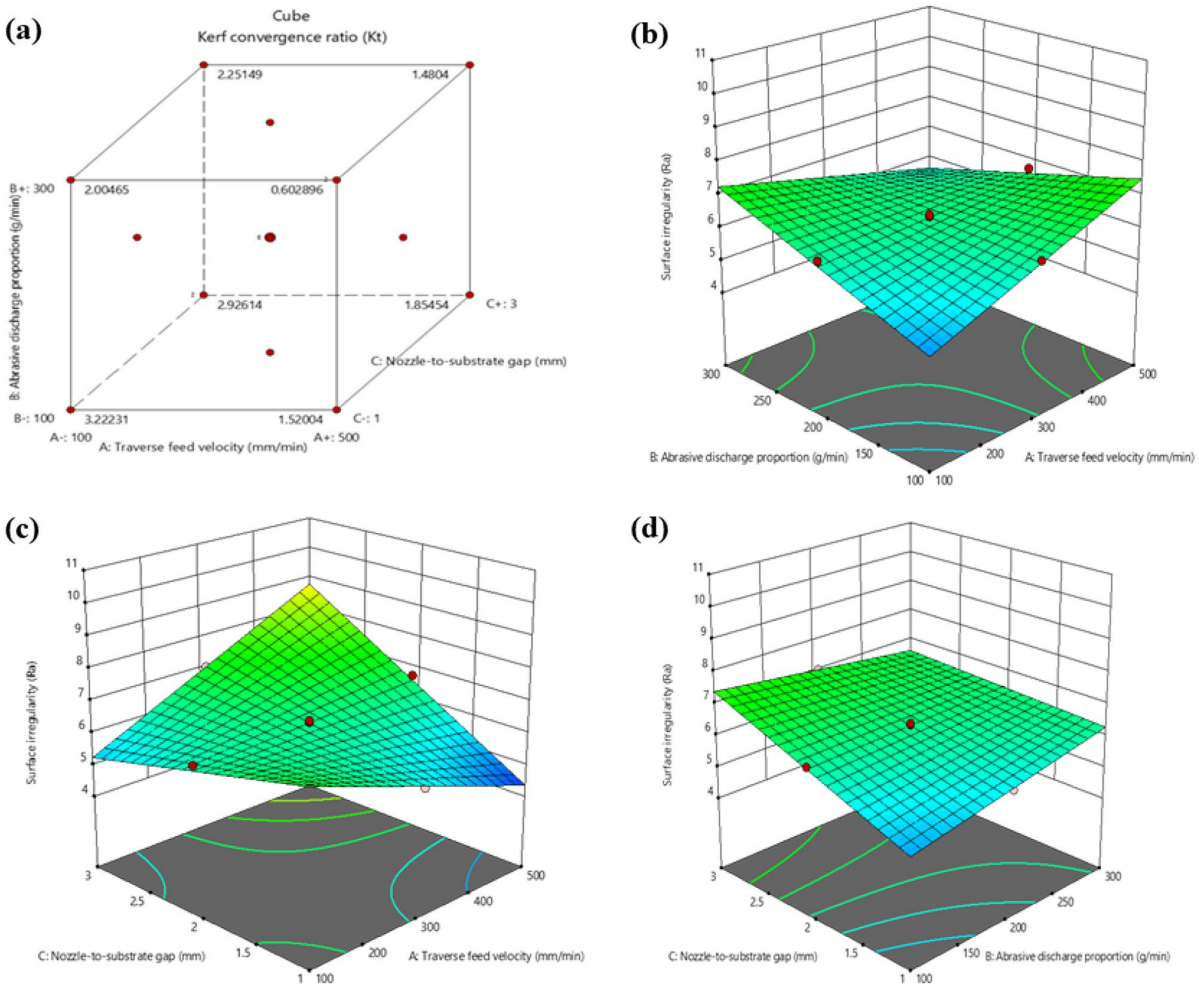
Interaction between Surface irregularity and machining variables **(a)** Cube plot (**b**) 3D surface plot with predictors Abrasive discharge proportion and traverse feed velocity for Surface irregularity (**c**) 3D surface plot with predictors traverse feed velocity and Nozzle-to-substrate gap for Surface irregularity (**d**) 3D surface plot with predictors Abrasive discharge proportion and Nozzle-to-substrate gap for Surface irregularity.

Figure 7 demonstrates the impact of different machining variables on Surface irregularity. Increasing traverse feed velocity from 100 to 500 mm/min reduces Surface irregularity because the nozzle can cover a larger area in a shorter time, resulting in a smoother surface. However, reducing traverse feed velocity can result in a rougher surface due to less solid removal from the workpiece. A faster workpiece movement leads to fewer impacts per unit area and a smoother surface. Furthermore, increasing the Abrasive discharge proportion can reduce Surface irregularity by removing more material from the workpiece surface per unit of time. However, suppose the Nozzle-to-substrate gap is too small. In that case, the high pressure and velocity of the waterjet can lead to a rough surface irregularity. In contrast, a Nozzle-to-substrate gap that is too large can result in a smoother surface irregularity due to less impact on the workpiece.

### Impact of machining variables on Kerf convergence ratio (K_t_) of H3 Laminate

Equation (6) depicts the mathematical relationship between the Kerf convergence ratio and the machining variables for H3 laminate containing 6 wt% silicon carbide filler.

6 $${K}_{t}= +0.000412+0.004148 {V}_{t}+0.003701 {m}_{a}+0.324425 S-0.000013 \times {V}_{t}\times {m}_{a}-0.000691\times {V}_{t}\times S+0.000739\times {m}_{a}\times S$$

Equation (6) shows that for 6 wt% silicon carbide filler particles on fiber/metal laminate, Kerf convergence ratio is positively affected by traverse feed velocity, Abrasive discharge proportion, Nozzle-to-substrate gap, and the interaction between Abrasive discharge proportion and Nozzle-to-substrate gap, while the interaction between traverse feed velocity and Abrasive discharge proportion and traverse feed velocity and Nozzle-to-substrate gap has a negative effect. The 2FI model is used to analyze the relationship between Kerf convergence ratio, traverse feed velocity, Abrasive discharge proportion, and Nozzle-to-substrate gap, and it is considered significant. Table 5 shows the significant influence and interaction of machining variables on the Kerf convergence ratio, with R^2^ and adjusted R^2^ being 0.9675 and 0.9526 for H3 laminate, indicating a strong significance between the variable response variables. The model for H3 laminates has a *p*-value below 0.05, indicating that the statistical model is significant. For H3 laminate, traverse feed velocity and Abrasive discharge proportion individually contribute more than 30% and 15%, respectively, accounting for nearly two-thirds of the percentage on the Kerf convergence ratio. Other interactions between machining variables and individual parameter contributions have minimal impact on determining the Kerf convergence ratio. This contribution level suggests that traverse feed velocity and Abrasive discharge proportion are crucial factors in determining the Kerf convergence ratio for H3 laminate.

**Figure 8:**
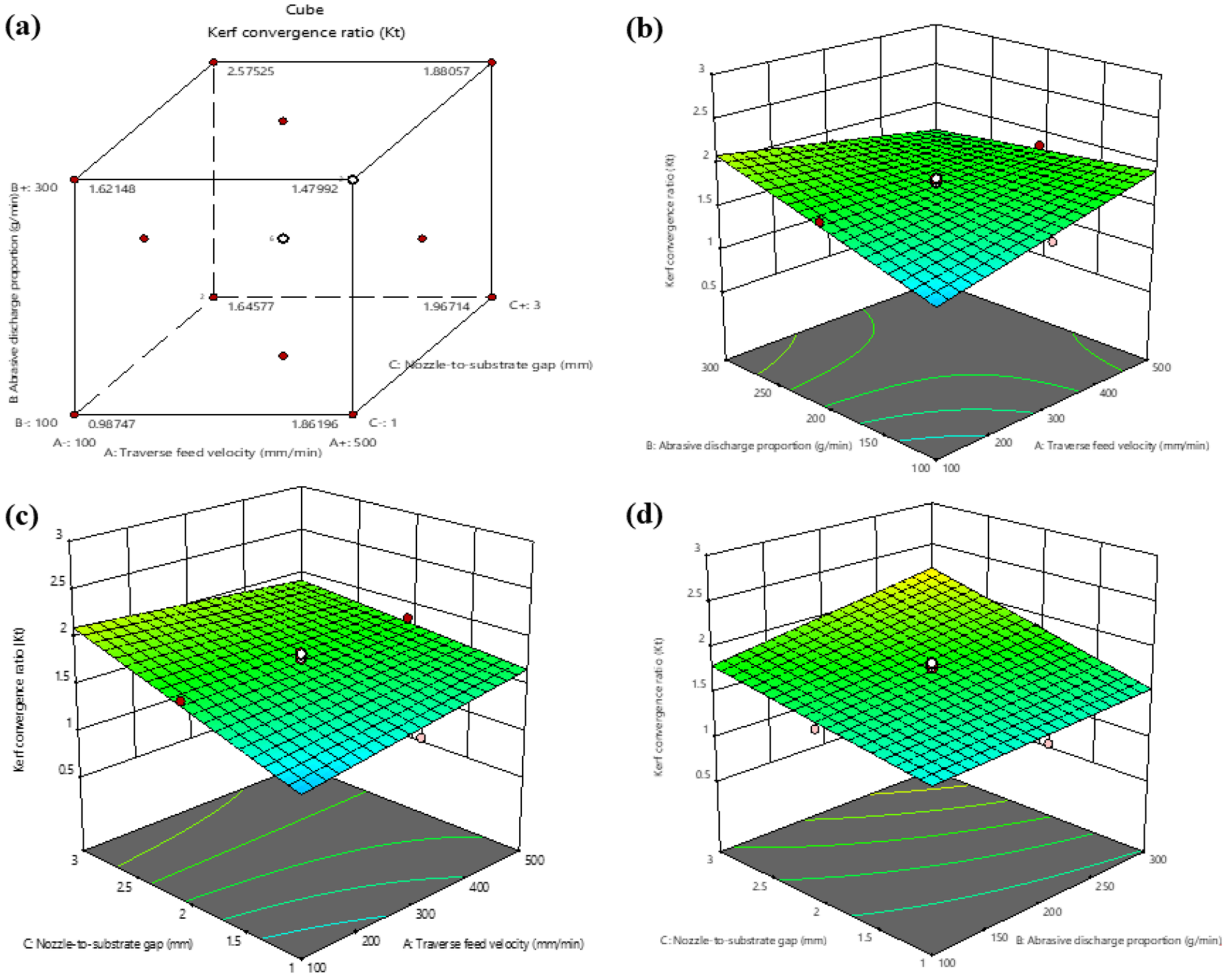
Interaction between Kerf convergence ratio (K_t_) and machining variables (**a**) Cube plot (**b**) 3D surface plot with predictors Abrasive discharge proportion and traverse feed velocity for Kerf convergence ratio (**c**) 3D surface plot with predictors traverse feed velocity and Nozzle-to-substrate gap for Kerf convergence ratio (**d**) 3D surface plot with predictors Abrasive discharge proportion and Nozzle-to-substrate gap for Kerf convergence ratio.

Figure 8 depicts that reducing traverse feed velocity leads to a decrease in the Kerf convergence ratio. This is because a slower traverse feed velocity allows more time for the abrasive particles to wear away the material, creating a wider kerf with a lower taper ratio. A more incredible traverse feed velocity, on the other hand, results in a narrower kerf with a larger taper ratio because the abrasive particles get less time to degrade the material. Additionally, a slower traverse feed velocity helps the abrasive particles maintain a more consistent impact angle with the material, contributing to a lower taper ratio. Moreover, an increase in the Abrasive discharge proportion leads to a decrease in the Kerf convergence ratio, according to Fig. 8. This is because the abrasive particles carry more impact energy at a higher feed rate, resulting in greater material removal from the centre of the kerf, leading to a narrower kerf and a lower taper ratio. Conversely, if the Abrasive discharge proportion is decreased, the impulse energy of the abrasive elements on the material is reduced, resulting in a lesser amount of material being removed from the centre of the kerf. This leads to a wider kerf with a higher taper ratio.

Additionally, higher ma levels tend to remove the material more uniformly, contributing to a lower taper ratio. The Nozzle-to-substrate gap increases the Kerf convergence ratio since the angle at which the abrasive particles hit the workpiece becomes more oblique as the distance between the nozzle and the workpiece increases. As a result, the particles remove more material from the edges of the kerf and less from the center, leading to a greater taper. Furthermore, the increase in the Nozzle-to-substrate gap results in a decrease in the velocity of the abrasive particles, which further contributes to the increase in the Kerf convergence ratio.

### Impact of machining variables on H3 Laminate Surface irregularity (R_a_)

Equation (7) mathematically represents the connection between the considered machining variables and Surface irregularity for the H3 laminate with 6 wt% silicon carbide filler particles.

7 $${R}_{a}= +5.14141-0.005669 {V}_{t}+0.033005 {m}_{a}-1.01864 S-0.000058 \times {V}_{t}\times {m}_{a}+0.009744\times {V}_{t}\times S-0.006782\times {m}_{a}\times S$$

According to Eq. (7), for fiber/metal laminates with 6 wt% silicon carbide filler particles, the Abrasive discharge proportion and the interaction of traverse feed velocity and Nozzle-to-substrate gap positively affect Surface irregularity. In contrast, traverse feed velocity and Nozzle-to-substrate gap alone have a negative impact. The 2FI model is the preferred method for examining the connection between Surface irregularity, traverse feed velocity, Abrasive discharge proportion, and Nozzle-to-substrate gap. The model is significant, as shown in Table 5, which displays the significant outcomes and interactions of the machining variables on Surface irregularity. The R^2^ and adjusted R^2^ values are 0.9841 and 0.9768, respectively, for H3 laminate, indicating a strong significance between the variable response variables. The statistical model is significant for H3 laminates, with a *p*-value of less than 0.05. For H3 laminate, the Nozzle-to-substrate gap alone contributes almost 50% to the determination of Surface irregularity, while the interaction of traverse feed velocity and Abrasive discharge proportion contributes 35%. Other interactions between machining variables and individual parameter contributions have minimal impact on determining Surface irregularity. This level of contribution indicates that the Nozzle-to-substrate gap is a crucial factor in determining Surface irregularity for H3 laminate.

**Figure 9:**
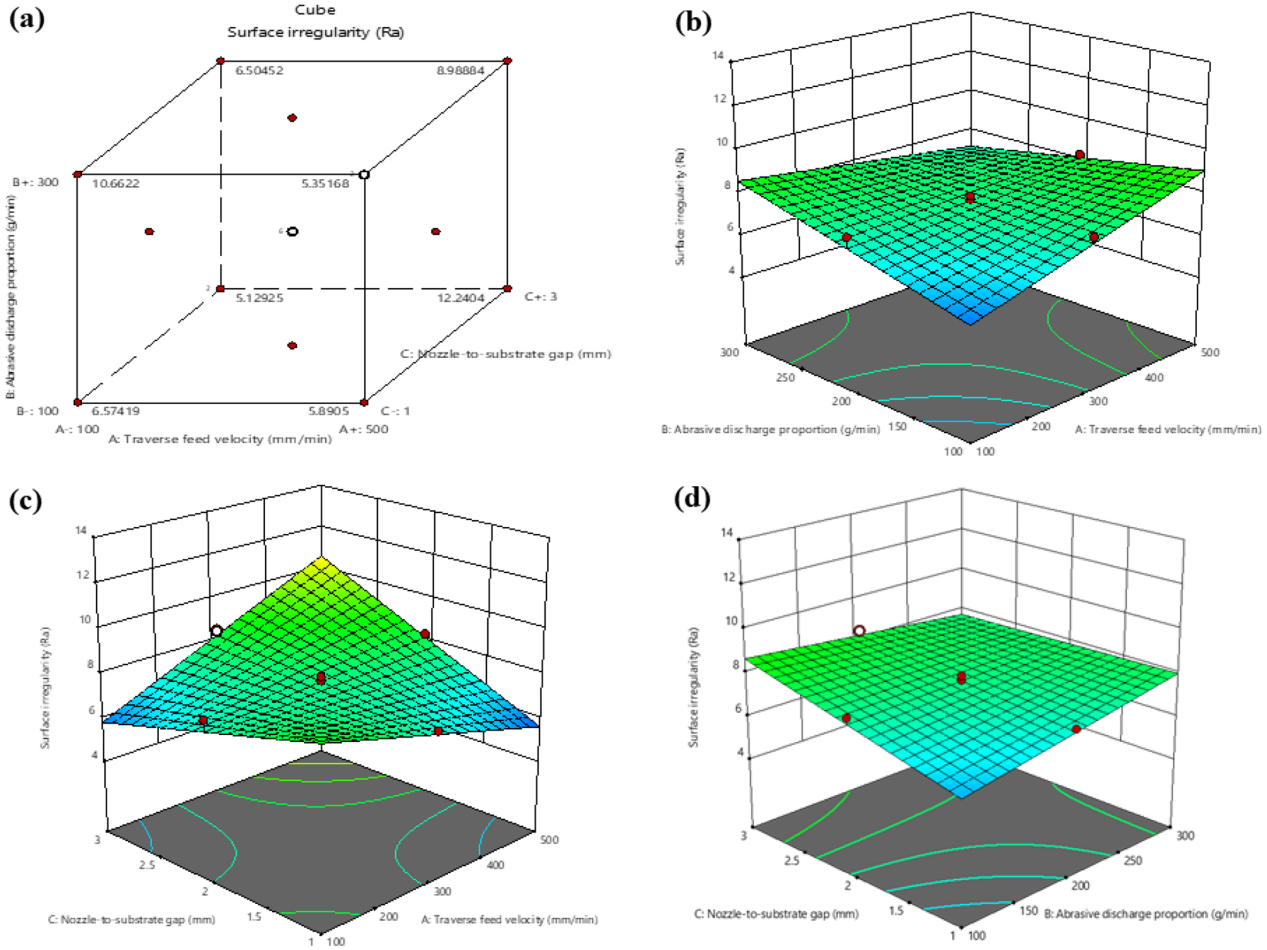
Interaction between surface irregularity and machining variables (**a**) Cube plot (**b**) 3D surface plot with predictors Abrasive discharge proportion and traverse feed velocity for Surface irregularity (**c**) 3D surface plot with predictors traverse feed velocity and nozzle-to-substrate gap for Surface irregularity (**d**) 3D surface plot with predictors Abrasive discharge proportion and Nozzle-to-substrate gap for Surface irregularity.

In Fig. 9, it is apparent that Surface irregularity increases as the traverse feed velocity increases. When the traverse feed velocity rises, the waterjet pressure and Abrasive discharge proportion must be modified to maintain a constant cutting velocity. If the traverse feed velocity is increased without adjusting the waterjet pressure and abrasive flow rate, the cutting velocity will also increase, resulting in a rougher surface irregularity. Additionally, as the traverse feed velocity increases, the abrasive particles in the waterjet may not have sufficient time to erode the material thoroughly, causing a rougher Surface irregularity. The abrasive particles will also impact the material at a higher velocity, leading to more micro-fractures and, consequently, a rougher surface irregularity.

The diagram in Fig. 9 demonstrates that a decrease in the nozzle-to-substrate gap produces high pressure and velocity of the water jet, ensuing in an uneven surface irregularity on the laminate. On the other hand, an increase in the nozzle-to-substrate gap causes a lower impact on the workpiece, resulting in a smoother surface irregularity. As the Nozzle-to-substrate gap increases, the jet disperses more widely before striking the surface, lowering the kinetic energy density at impact and leading to a coarser surface. Additionally, this jet dispersion results in a lower concentration of abrasive particles. Conversely, a smaller nozzle-to-substrate gap results in a better-machined surface irregularity of the laminate.

### Optimization of machining variables

**Figure 10:**
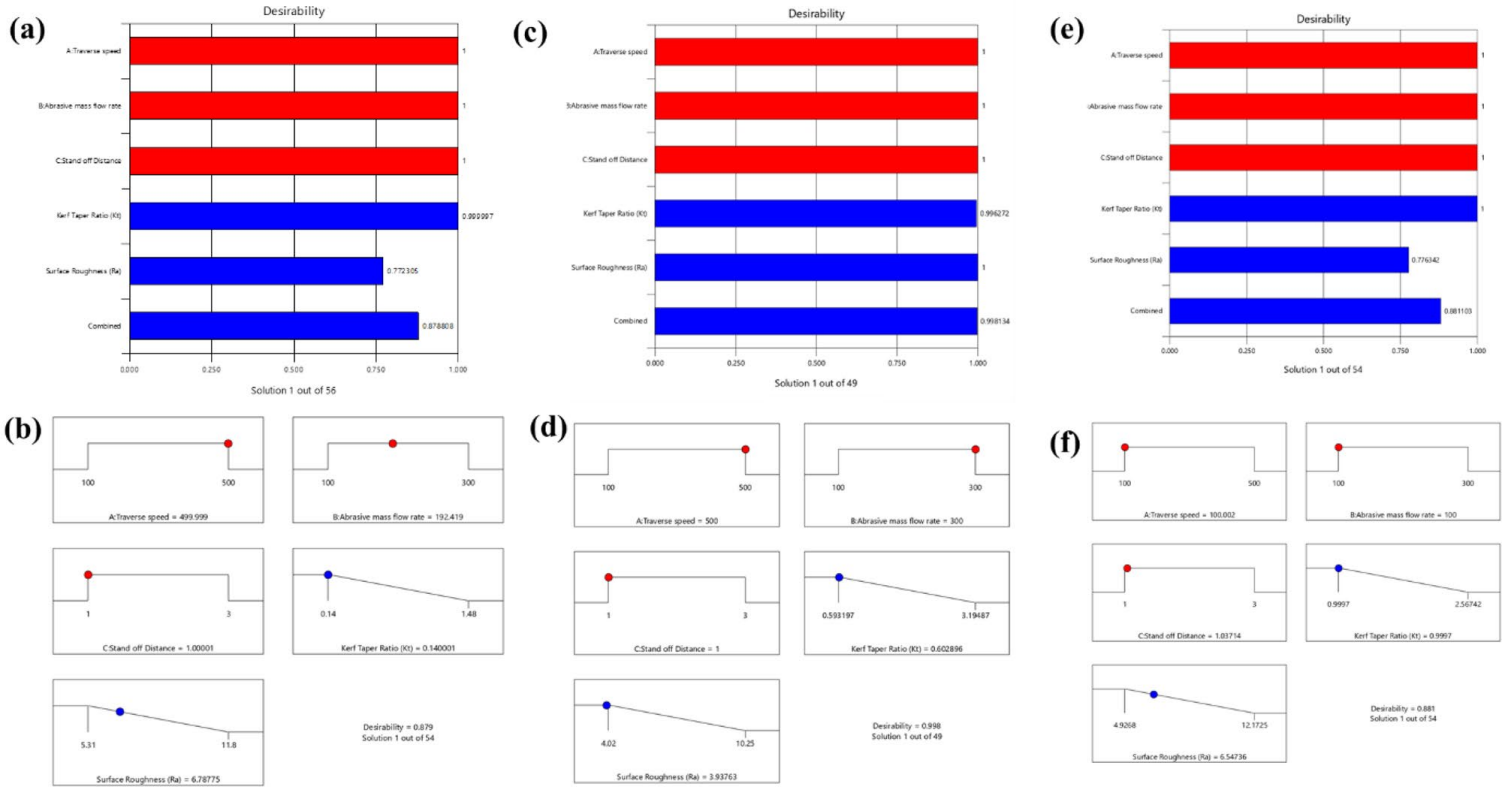
(**a**) Bar chart showing the level of desirability for H1 laminate (**b**) Demonstration of the ramp plot for overall desirability for H1 laminate (**c**) Bar chart showing the level of desirability for H2 laminate (**d**) Demonstration of the ramp plot for overall desirability for H2 laminate (**e**) Bar chart showing the level of desirability for H3 laminate (**f**) Demonstration of the ramp plot for overall desirability for H3 laminate.

Table 7 illustrates the restrictions on input, process variables, response targets, and top outcomes for H1, H2, and H3 laminates. For H1 and H2 laminates, the ideal process variables for the specific targets are projected to be a traverse feed velocity of 500 mm/min, an Abrasive discharge proportion of 192.417 g/min & 300 g/min, and a Nozzle-to-substrate gap of 1 mm, with a maximum desirability of 0.879 and 0.998, respectively. The most appealing outcome of 0.881 is achieved for the H3 laminate when the traverse feed velocity is set at 100 mm/min, the Abrasive discharge proportion is 100 g/min, and the Nozzle-to-substrate gap is 1 mm. The ramp plot for the desired input process parameter optimizes the bar histogram plot for the desirability of H1, H2, and H3 laminates, as demonstrated in Fig. 10.

**Table 7:** Constraints and Optimal Solutions.

Variables	Objective	Lower bound	Upper bound	Optimal Solution	Lower bound	Upper bound	Optimal Solution	Lower bound	Upper bound	Optimal Solution
		H1 Laminate			H2 Laminate			H3 Laminate		
A: Traverse feed velocity	Is in Range	100	500	500	100	500	500	100	500	100
B: Abrasive discharge proportion	Is in Range	100	300	192.417	100	300	300	100	300	100
C: Nozzle-to-substrate gap	Is in Range	1	3	1	1	3	1	1	3	1
Kerf convergence ratio	Minimize	0.14	1.48	0.14004	0.5932	3.1949	0.603	0.9997	2.5675	0.999
Surface irregularity	Minimize	5.31	11.8	6.788	4.02	10.25	3.938	4.9268	12.1725	6.5473

### Model validation

**Figure 11:**
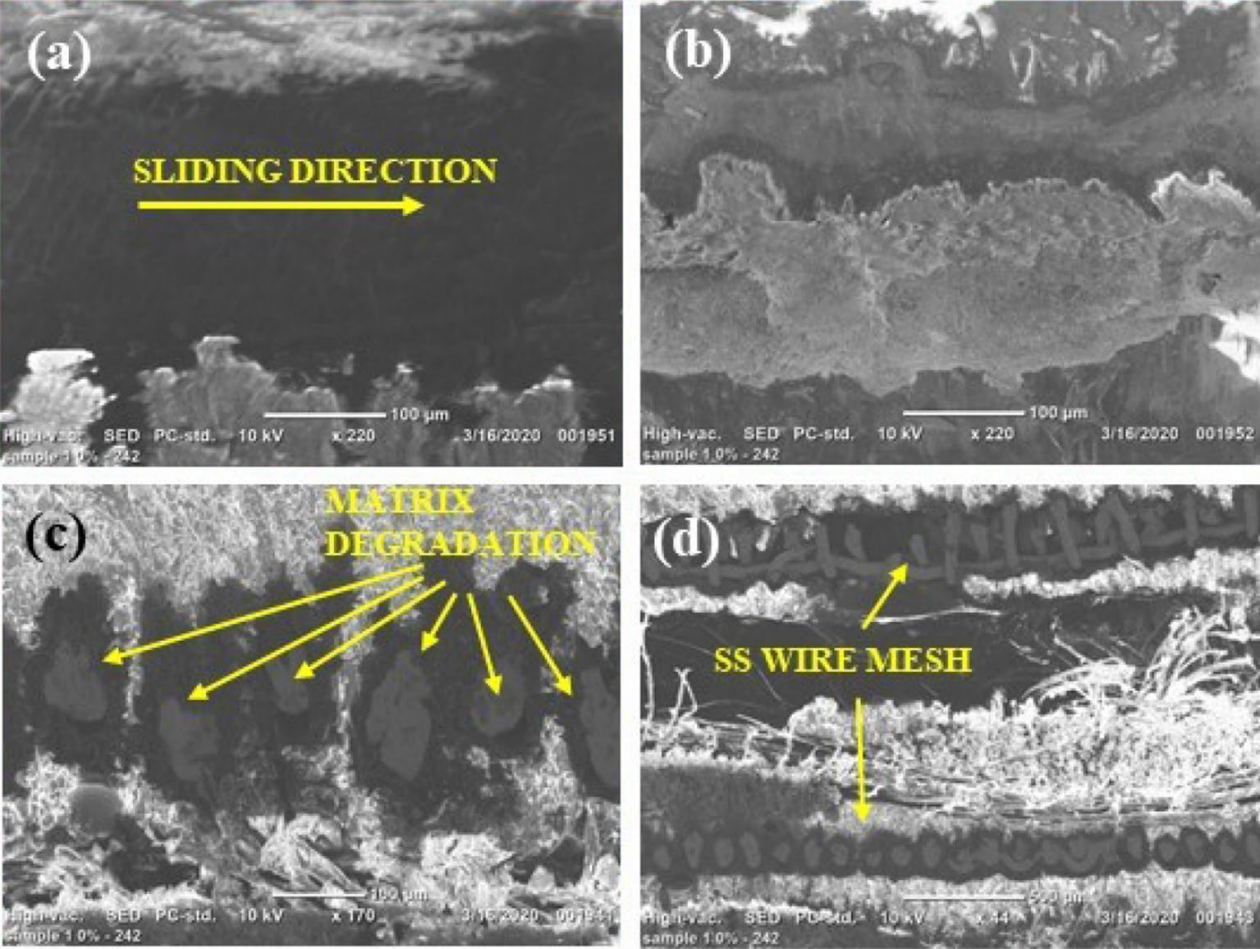
Scanning electron micrographs of H1 laminate (0 wt% SiC).

Three fresh trials were conducted to independently confirm the H1, H2, and H3 laminate models' accuracy, employing optimal cutting variables. The average surface irregularity and Kerf convergence ratio were computed, and the models' precision was assessed by measuring the percentage error (Fig. 11). The vibration during the machining process was likely responsible for the discrepancies in the results, which affected the measurement process. However, since the error was under 5%, it can be concluded that there is a robust relationship between the anticipated and experimental data (Table 8).

**Table 8:** Comparison of actual and predicted outcomes when process variables are at their optimal values.

Output responses	Predicted	Observed	Error (%)	Predicted	Observed	Error (%)	Predicted	Observed	Error (%)
Kerf convergence ratio (K_t_)	0.1400	0.14335	2.39	0.6029	0.6235	3.42	0.999	1.0195	1.96
Surface irregularity (R_a_)	6.788	6.993	3.02	3.9377	4.096	4.02	6.5473	6.8301	4.32SE

#### SEM micrographs

**Figure 12:**
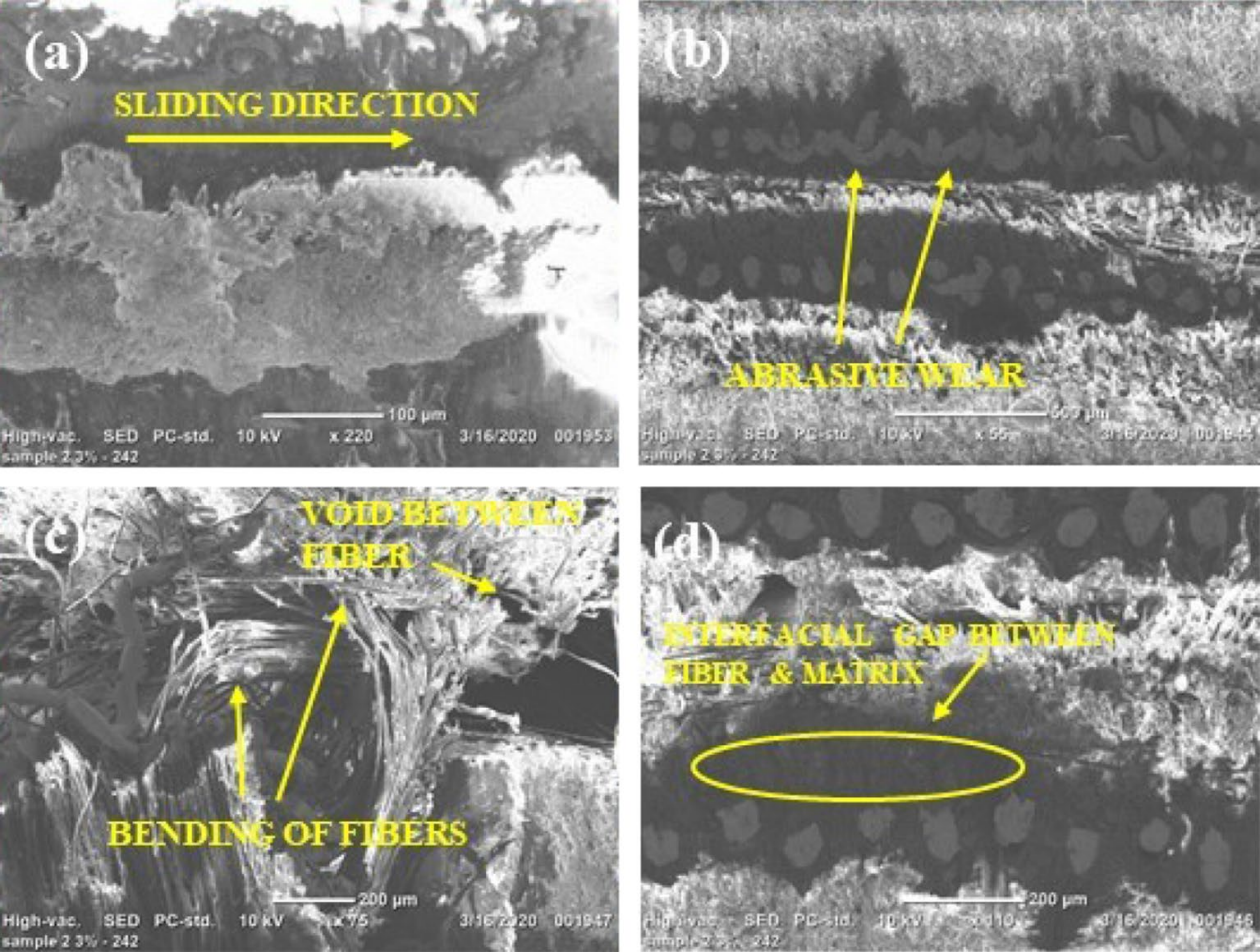
Scanning electron micrographs of H2 laminate (3 wt% SiC).

**Figure 13:**
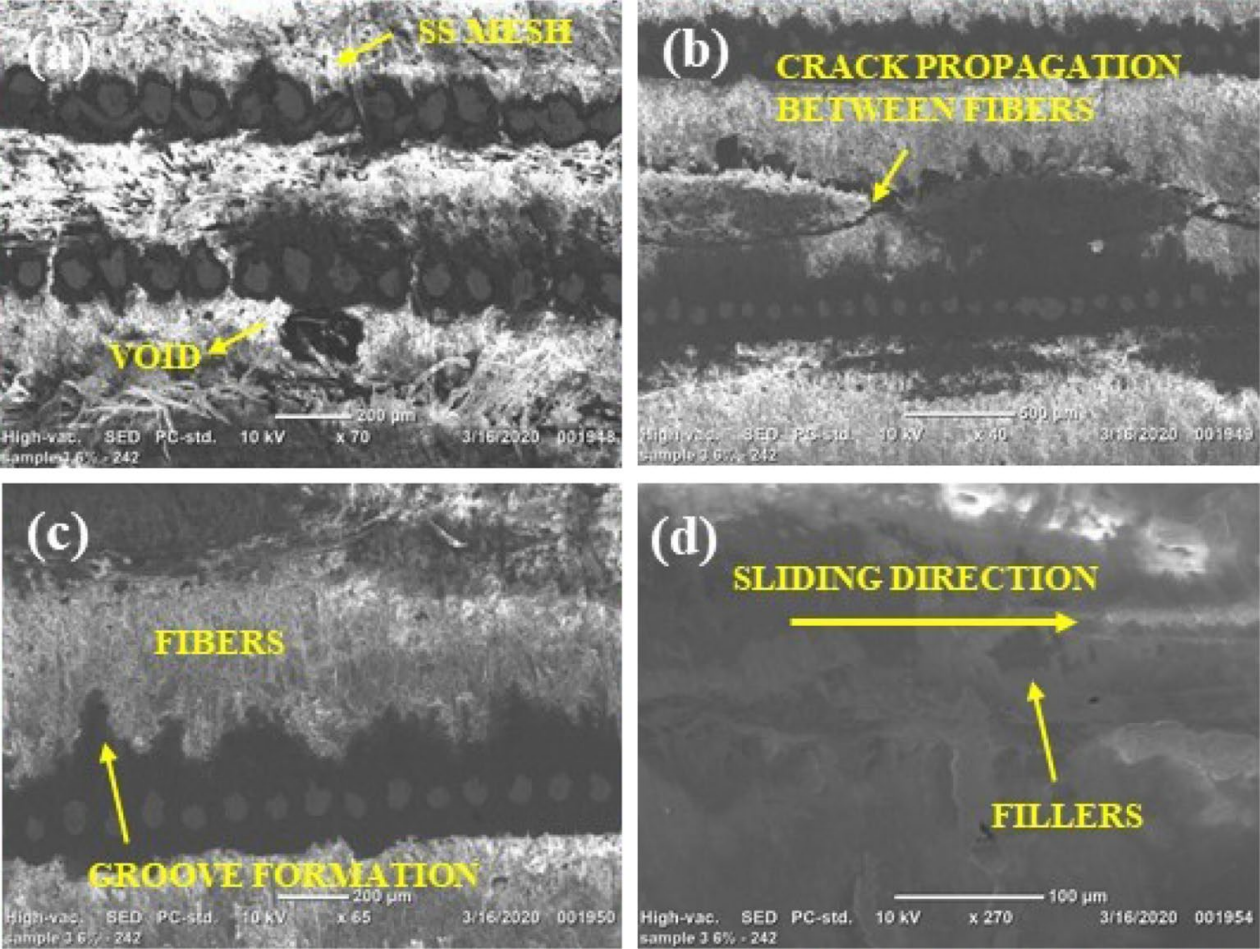
Scanning electron micrographs of H3 laminate (6 wt% SiC).

The scanning electron microscopy images of abrasive waterjet machined (AWJM) fiber/metal hybrid composites provide insights into the material removal process and the surface characteristics of the machined parts. Some inferences drawn from SEM images of AWJM fiber/metal hybrid composites include the following:

- The scanning electron microscope pictures reveal surface properties of machined components, such as the presence of residual abrasive particles, the size and arrangement of surface features, and the overall texture. The analysis of the images indicates that the addition of silicon carbide fillers leads to a smoother surface with a more uniform morphology, as evidenced by Figs. 12 and 13, in comparison to composites that do not have silicon carbide fillers, as shown in Fig. 11.
- The scanning electron microscope images furnish details regarding the material removal mechanism in abrasive water jet machining, including any signs of melting, vaporization, or abrasion marks on the surface. This knowledge aids in comprehending how the abrasive waterjet eliminates the material and how the machining process affects it. These images are also employed to evaluate the surface quality of the machined components, detecting any flaws or flaws that may have arisen during the machining procedure.
- The scanning electron microscope images also furnish details regarding the microstructural characteristics of the machined components, such as the orientation and distribution of fibers in the composite laminate, interfacial bonding between the fibers and the matrix, and the uniformity of the material overall.
- The scanning electron microscope images demonstrate that the composites with silicon carbide fillers have a more significant density of abrasive marks on their surface, indicating a greater abrasion resistance. Adding silicon carbide fillers to fiber/metal hybrid composites can enhance their mechanical properties by improving the interfacial connection between the fibers and the matrix. Scanning electron microscope images reveal that composites with silicon carbide fillers exhibit a more uniform distribution and orientation of fibers and a higher bonding density between the fibers and the matrix.

## Conclusion

This study employs Abrasive Water Jet Machining (AWJM) to optimize fiber-metal composites that contain 0%, 3%, and 6% silicon carbide fillers using Response Surface Methodology (RSM). The aim is to find the ideal responses for the composites based on their desirability (D) values and to address the multi-response parameter optimization in AWJM. The following inferences are made from the data:

Regarding the H1 laminate, It was determined that its traverse feed velocity has the greatest effect on both the cutting angle and the surface irregularity. In the case of H2 laminate, the traverse feed velocity and Abrasive discharge proportion significantly impact the Kerf convergence ratio. The kerf convergence ratio of the H3 laminate is most significantly affected by the Nozzle-to-substrate gap, traverse feed velocity, and Abrasive discharge proportion.

Based on the computations, the optimal cutting variables for H1 laminate consist of a traverse feed velocity of 500 mm/min, an Abrasive discharge proportion of 192.417 g/min, and a Nozzle-to-substrate gap of 1 mm. For H2 laminate, the recommended variables are a traverse feed velocity of 500 mm/min, an Abrasive discharge proportion of 300 g/min, and a Nozzle-to-substrate gap of 1 mm. Finally, for H3 laminate, the most effective cutting conditions are a traverse feed velocity of 100 mm/min, an Abrasive discharge proportion of 100 g/min, and a Nozzle-to-substrate gap of 1 mm.

The interactions between variables have a noticeable impact on the responses, and the model's findings align with the experimental results with a 95% level of certainty. The degree of error in predicting response variables is in line with investigational outcomes.

The examination of the cut surface with a scanning electron microscope showed that when the traverse feed velocity was higher, fiber pull-outs, matrix washout, and delamination were less common for H1 and H2 laminates. Additionally, these issues were even less common for the H3 laminate, regardless of the type of laminate being examined.

## Data Availability

All data generated or analysed during this study are included in this published article.
